# Reversible conformational change in herpes simplex virus glycoprotein B with fusion-from-without activity is triggered by mildly acidic pH

**DOI:** 10.1186/1743-422X-7-352

**Published:** 2010-12-01

**Authors:** Carlos R Siekavizza-Robles, Stephen J Dollery, Anthony V Nicola

**Affiliations:** 1 Department of Microbiology and Immunology, Virginia Commonwealth University School of Medicine, 1101 East Marshall Street, Richmond, Virginia 23298-0678, USA

## Abstract

**Background:**

The pre-fusion form of the herpes simplex virus (HSV) fusion protein gB undergoes pH-triggered conformational change *in vitro *and during viral entry (Dollery et al., J. Virol. 84:3759-3766, 2010). The antigenic structure of gB from the fusion-from-without (FFWO) strain of HSV-1, ANG path, resembles wild type gB that has undergone pH-triggered changes. Together, changes in the antigenic and oligomeric conformation of gB correlate with fusion activity. We tested whether the pre-fusion form of FFWO gB undergoes altered conformational change in response to low pH.

**Results:**

A pH of 5.5 - 6.0 altered the conformation of Domains I and V of FFWO gB, which together comprise the functional region containing the hydrophobic fusion loops. The ANG path gB oligomer was altered at a similar pH. All changes were reversible. In wild type HSV lacking the UL45 protein, which has been implicated in gB-mediated fusion, gB still underwent pH-triggered changes. ANG path entry was inactivated by pretreatment of virions with low pH.

**Conclusion:**

The pre-fusion conformation of gB with enhanced fusion activity undergoes alteration in antigenic structure and oligomeric conformation in response to acidic pH. We propose that endosomal pH triggers conformational change in mutant gB with FFWO activity in a manner similar to wild type. Differences apart from this trigger may account for the increased fusion activity of FFWO gB.

## Introduction

Membrane fusion during enveloped virus entry is mediated by conformational change in viral fusion proteins. Herpesviruses are a paradigm for viral entry mediated by a multi-component fusion machinery. Herpesviral fusion and entry is further complicated by the likely requirement of multiple cellular cues. Herpes simplex virus (HSV) glycoproteins gB, gD, and gH-gL are necessary for entry and membrane fusion [1-3]. A cellular receptor for gD is essential for entry, but one or more additional cellular triggers is also required. There is mounting evidence for the critical, direct role of endosomal pH during HSV entry by endocytosis, which is the predominant entry pathway for HSV in many cell types including human epithelial cells [4,5]. Lysosomotropic agents, which elevate intravesicular pH, block HSV entry by trapping virions in endocytic compartments [4,6]. Pretreatment of isolated HSV particles with mildly acidic pH inactivates viral entry activity, which is a characteristic of viruses that are directly triggered by endosomal pH for fusion [4]. Low pH together with soluble gD-receptor triggers association of HSV with artificial membranes [7].

We recently demonstrated that gB present in virions, i.e., the pre-fusion form, undergoes conformational change in direct response to mildly acidic pH of 5.5 to 6.0, both *in vitro *and during viral entry into cells [8]. Low pH caused a specific change in the antigenic structure of the functional region of gB containing the hydrophobic, bipartite fusion loops. A similar range of mildly acidic pH caused a change in the oligomeric conformation of gB. Low pH triggered gB to become more hydrophobic, suggesting that membrane-interacting regions are revealed. Conformational changes in gB were reversible. Taken together, these findings support a model in which endosomal low pH serves as a cellular trigger for fusion by activating the fusion protein gB [8].

The product of the HSV UL45 gene is a non-glycosylated, membrane protein that is present in the virion envelope and is dispensable for viral entry via endocytic and non-endocytic cell entry pathways [9,10]. The role of the UL45 protein in the viral envelope is not known. HSV syncytium formation mediated by a Y854K mutation in the cytoplasmic tail of gB requires wild type UL45 [11].

Thus, UL45 may mediate fusion events during HSV infection through a functional interaction with gB.

Fusion-from-without (FFWO) is the rapid induction of cell fusion by virions in the absence of viral protein synthesis [12]. HSV-1 ANG path is a prototype FFWO strain. The combination of two amino acid mutations in gB, one in the ectodomain (V553A) and one in the cytoplasmic tail (A855V), confers FFWO activity to wild type HSV [13]. Virion-cell fusion during entry has been refractory to direct study. FFWO is a surrogate assay for fusion during entry because it parallels viral entry in several respects [14-16]. Importantly, the effector and target membranes for FFWO and entry are the same. Like entry, FFWO requires an appropriate gD-receptor in the target membrane. The efficiency of gD-receptor usage for FFWO correlates with the efficiency of entry mediated by the same receptor. Lastly, antibodies to gB and gD that block FFWO also neutralize virus entry. The pre-fusion form of gB with FFWO activity has an altered antigenic conformation relative to wild type gB [16]. Interestingly, the pre-fusion wild type gB undergoes conformational changes in these same antigenic sites upon exposure to low pH [8]. FFWO strains of HSV require endosomal low pH for entry in a cell-specific manner, similar to wild type [4,16]. However, FFWO itself occurs at neutral pH and is not enhanced by acidic pH (unpublished data). In this report, we investigate the relationship between pH-triggered conformation changes and fusion activity by analyzing the effect of pH on virion gB with FFWO activity.

## Results and discussion

**Figure 1:**
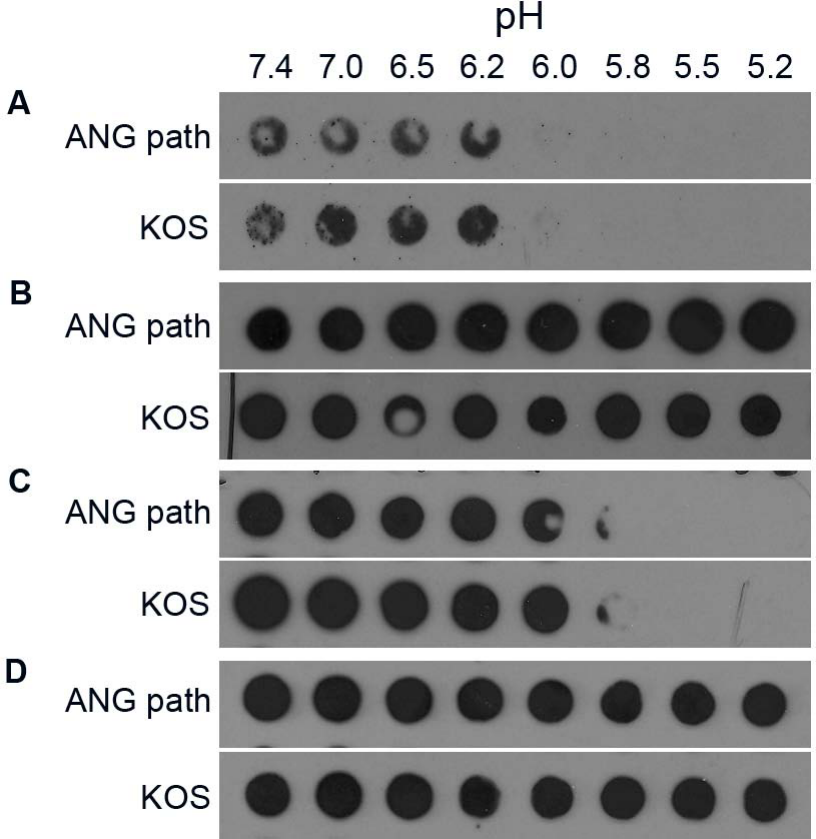
**Reactivity of gB-specific antibodies with HSV-1 ANG path virions treated with low pH**. Extracellular HSV-1 ANG path or KOS virions were treated for 5 min at 37°C with medium buffered to the indicated pHs and were blotted immediately to nitrocellulose membrane. Blots were probed at pH 7.4 with the gB-specific MAb (A) H126, (B) SS10, (C) SS106 or (D) H1817 followed by horseradish peroxidase conjugated goat secondary antibody. The exposures shown highlight the pH thresholds.

The H126 epitope, which is in the fusion domain of gB, and the DL16 epitope, which is specific for the gB trimer, are diminished in both FFWO ANG path gB and low pH-treated, wild type gB [8,16]. This led to the suggestion that changes in these epitopes are related to fusion function. We theorized that if the pre-fusion antigenic and oligomeric conformation of ANG path gB is responsible for enhanced fusion activity, then it might undergo altered conformational change in response to low pH. HSV-1 ANG path virions were exposed to a range of pHs, blotted directly to nitrocellulose, and then antibody binding was measured at neutral pH. MAbs H126 (Figure 1A) and SS106 (Figure 1C) displayed diminished binding to gB from ANG path virions that had been treated at pH < 6.2 or 6.0. These MAbs recognize Domains I and V, respectively, which together make up Functional Region 1 of gB [17]. MAbs SS10 (to Domain II) (Figure 1B) and H1817 (to Domain VI) (Figure 1D) displayed unaltered binding to acid-treated virions, indicating that pH does not cause a global change in ANG path gB conformation. For the epitopes tested, the antigenic conformation of the highly fusogenic mutant gB from strain ANG path was altered by low pH in a manner similar to wild type KOS (Figure 1; [8]).

**Figure 2:**
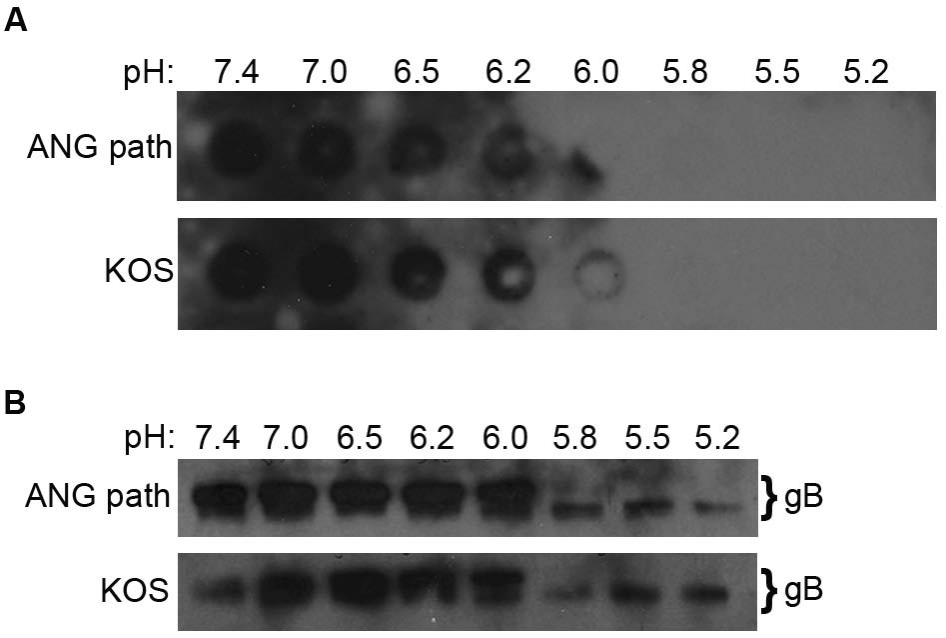
**Effect of low pH treatment on the oligomeric conformation of mutant gB with elevated fusion activity**. (A) Reactivity of oligomer-specific monoclonal antibody DL16 with low pH-treated HSV-1 ANG path virions. As in Figure 1, virions were treated with pHs ranging from 7.4 to 5.2, and were blotted to membrane. Blots were probed at neutral pH with trimer-specific MAb DL16. (B) The indicated virions were treated with pHs ranging from 7.4 to 5.2, solubilized with 1% SDS, and then analyzed by "native" PAGE. Immunoblots were probed with gB-specific polyclonal antibody. Panel shows the major gB species, which migrate slower than a 181 kilodalton protein standard (not shown).

To test the effect of acid pH on the oligomeric conformation of ANG path gB, we first probed acid-treated ANG path virions with oligomer-specific MAb DL16. DL16 displayed diminished binding to ANG path that had been treated at pH < 6.2 (Figure 2A), suggesting that this oligomer-specific epitope in FFWO gB is altered by mildly acidic pH. Secondly, we took advantage of an experimentally useful characteristic of HSV gB: Oligomers of gB are not disrupted by 1% SDS treatment, as measured by the migration of oligomeric species on native PAGE [8]. When gB is first exposed to low pH, its oligomeric structure then becomes susceptible to disruption by 1% SDS. Treatment of ANG path with pH 7.4 followed by 1% SDS yielded a range of oligomeric species of > 181 kDa (Figure 2B). However, pretreatment with pH < 6.0 followed by 1% SDS reduced the number of FFWO gB species detected (Figure 2B). The highest-molecular-weight forms seemed to disappear, leaving a predominant detectable oligomeric species of lower molecular weight (Figure 2B). This suggests that low pH alters the oligomeric structure of highly fusogenic gB, making it more sensitive to disruption by SDS. With decreasing pH, there was an apparent decrease in detection of gB-reactive species. One explanation is that monomers are detected only weakly relative to oligomers under standard native PAGE analysis (data not shown). Alternately, upon activation by pH, gB may become part of a larger complex that does not enter the native gel. Notably, the total amount of gB detected by dot blot does not change upon exposure to mildly acidic pH. Together, the two approaches suggest that the pre-fusion oligomeric forms of FFWO and wild type gBs undergo changes upon exposure to acidic pH.

**Figure 3:**
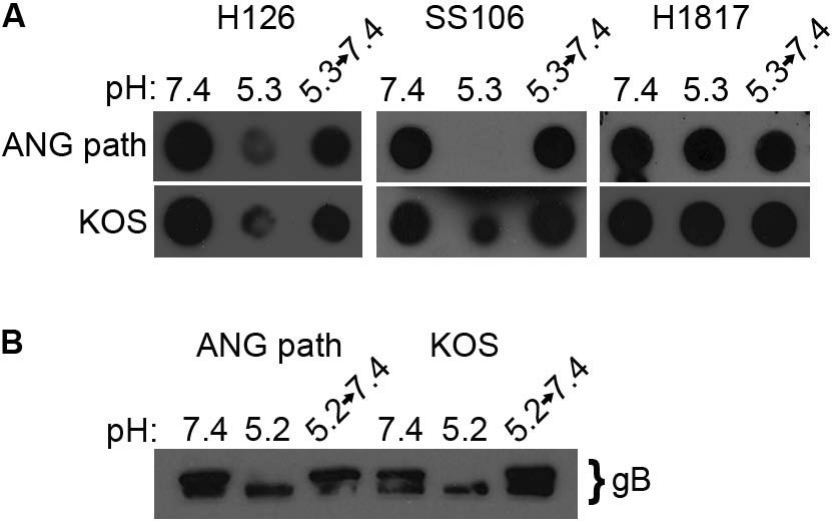
**Reversibility of pH-induced conformational changes in ANG path gB**. (A) HSV-1 ANG path or KOS virions were treated with medium buffered to pH 7.4 or 5.3. For the indicated samples, pH was neutralized back to 7.4 for 5 min at 37°C. Virions were blotted immediately to nitrocellulose. Membranes were probed at neutral pH with antibodies H126, SS106 or H1817 followed by horseradish peroxidase conjugated secondary antibody. The exposures shown document the reversibility of reactivity. (B) ANG path or KOS virions were treated with pH 7.4 or 5.2. Where indicated virions were neutralized back to pH 7.4. Samples were treated with 1% SDS, and then analyzed by "native" PAGE. Immunoblots were probed for detection of gB.

The pH-triggered conformational changes in HSV gB and other class III fusion proteins are reversible [8,18,19]. Reversibility may allow class III proteins to avoid non-specific activation during transport through the low pH environment of the secretory pathway. We tested whether acid-induced changes in the highly fusogenic ANG path gB were reversible. ANG path virions were treated at pH 5.3 to trigger conformational change, and were then adjusted back to pH 7.4 prior to blotting to nitrocellulose. Reactivity to MAbs H126 and SS106 was partly recovered relative to virions that received pH 5.3 treatment only (Figure 3A), suggesting pH-triggered alterations in the antigenic structure of FFWO gB are reversible. Control MAb H1817 reacted similarly with ANG path that had been subjected to each of the different pH conditions (Figure 3A). To extend the findings of reversibility, ANG path virions were incubated at pH 5.2, reneutralized to pH 7.4, and then 1% SDS was added (Figure 3B). High molecular weight, oligomeric forms of gB were detected that were similar to those of gB that had been kept at pH 7.4. This suggests that low pH-induced changes in the oligomeric structure of ANG path gB are reversible.

**Figure 4:**
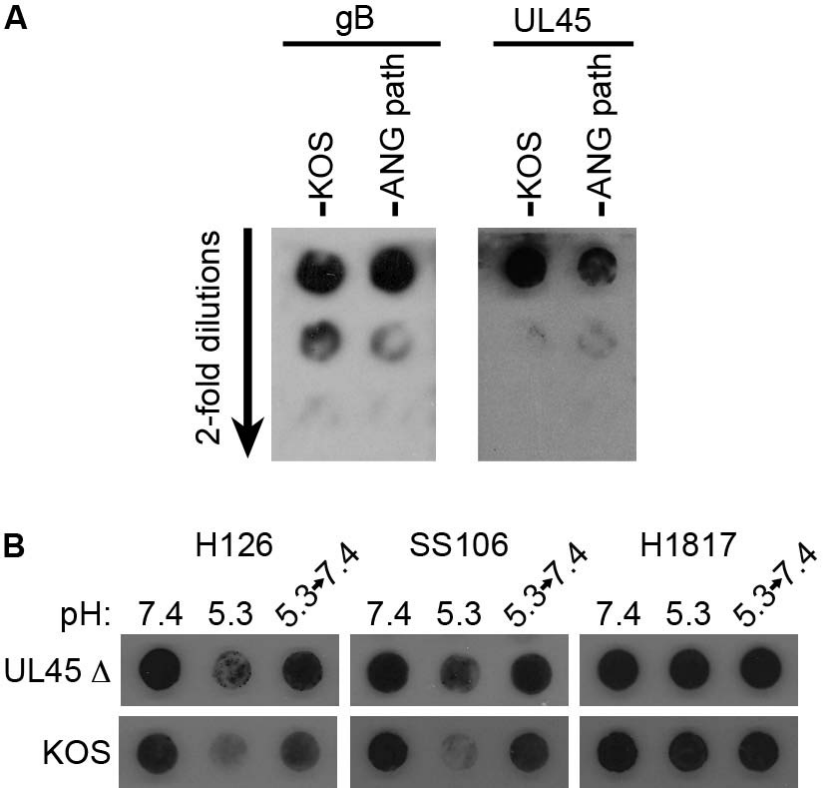
**Role of HSV-1 UL45 protein in the pH-triggered gB conformation change**. (A) UL45 protein content of HSV-1 ANG path virions. Two-fold dilutions of HSV-1 KOS or ANG path were blotted to nitrocellulose membrane. Blots were probed with polyclonal antibody specific for gB or UL45. (B) Reversible, pH-dependent conformational change in gB from virions lacking the UL45 protein. HSV-1 KOS UL45 D or KOS wild type virions were treated with pH as indicated in the legend to Figure 3A. Conformational change in gB was probed with the indicated antibodies.

The UL45 protein plays an undefined role in mediating cell-cell fusion. Although it is non-essential, it has been proposed to functionally interact with gB to regulate membrane fusion [11]. UL45 has no detectable effect on the conformation of the pre-fusion form of gB [10]. The UL45 protein was detected in ANG path virions to wild type levels (Figure 4A), indicating its presence in virions containing FFWO gB. To address the influence of UL45 on pH-triggered conformational changes in gB, we analyzed a UL45-null mutant, HSV-1 KOS UL45 D [9] (kindly provided by Curtis Brandt, University of Wisconsin). MAbs H126 and SS106 displayed diminished reactivity with pH 5.3-treated UL45-null virions relative to virions kept at pH 7.4 (Figure 4B). Reactivity of MAb H1817 was unaffected. Thus, the detected, pH-induced antigenic changes in gB occur in the absence of UL45 protein. Further, the UL45 protein does not influence the reversibility of acid-triggered changes in gB (Figure 4B). A functional role for UL45 in the viral envelope remains to be defined. It is possible that the UL45 protein may influence fusion-associated conformational changes that are not detected by these assays.

**Figure 5:**
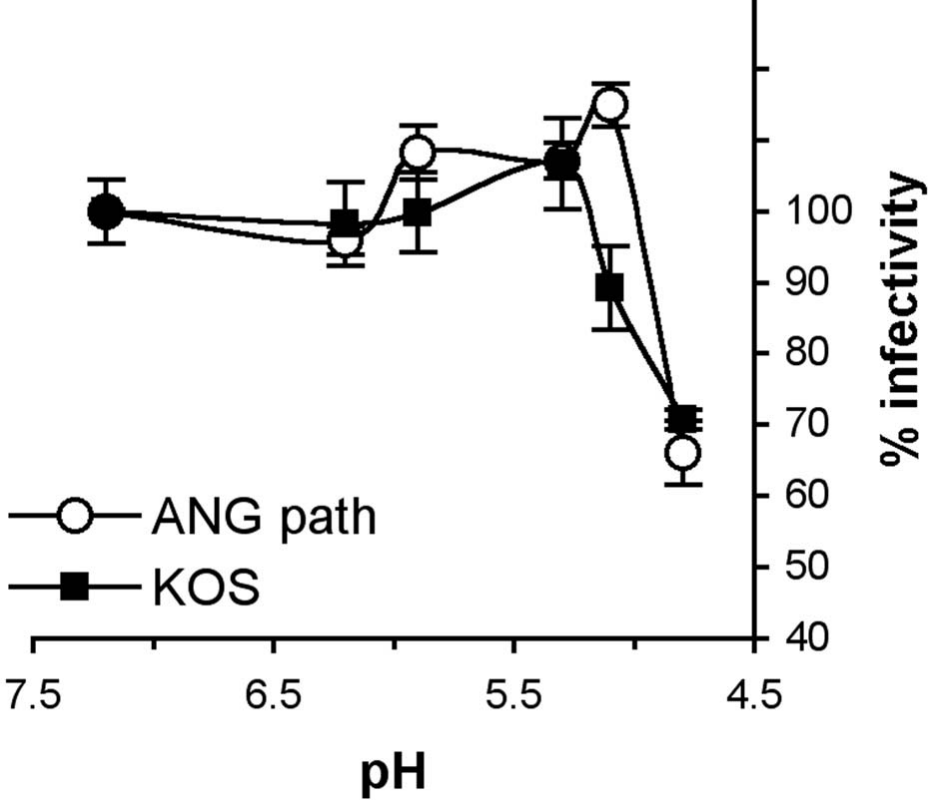
**Low pH inactivation of HSV-1 ANG path virions**. Samples of HSV-1 ANG path or KOS were adjusted to a range of pHs as shown, incubated at 37°C for 10 min, and then neutralized to pH 7.6. Treated virions were incubated with Vero cells, and plaque formation was measured as an indication of virus entry and infection. The infectivity of samples that were treated with pH 7.2 was defined as 100%. Data are means of quadruplicate wells with standard deviation.

Acid inactivation of virions is a feature of viruses that utilize pH-activated entry pathways. Low pH pre-treatment inactivates the entry of isolated herpes simplex virions in an irreversible and temperature-dependent manner [4]. This result, together with the findings that low pH alters gB structure, is consistent with the proposal that acid pH has a direct activating role in the fusion activity of HSV [8]. However, the virion target of low pH-mediated inactivation is not clear. To determine the susceptibility of HSV-1 with FFWO gB to inactivation, ANG path virions were treated at a range of pHs, adjusted to neutral pH, and then assayed for successful infection by plaque formation on Vero cells. A pH of < 5.3 was required to alter the entry activity of ANG path virions (Figure 5) under the conditions tested. Both ANG path and wild type strains were inhibited by ~ 30% when pretreated with pH 4.8 (Figure 5). Thus, the highly fusogenic form of gB present in ANG path virions did not alter virion inactivation. These results are consistent with low pH affecting HSV-1 ANG path in a manner similar to wild type (Figure 1 Figure 2 and Figure 3). Conformation changes in gB have a pH threshold of ~ pH 6 and are reversible, yet pH-induced inactivation of virions has a threshold of ~ 5 and is irreversible. There is no evidence that the detected changes in gB are responsible for inactivation. We propose that the mechanism of inactivation involves irreversible, pH-induced changes in HSV glycoproteins that are necessary for fusion [8].

Together the results indicate that conformational change in HSV-1 gB with FFWO activity is induced by pH ~ 5 to 6. We have proposed that low pH triggers the pre-fusion form of wild type gB, resulting in contact of its fusion loops with the target membrane [8]. Mildly acidic pH may have a similar effect on FFWO strains of HSV such as ANG path. The enhanced fusogenic activity of FFWO gB may manifest itself downstream of initial activation by pH, such as during refolding of gB when the two membranes are brought into apposition. Our current battery of assays likely does not detect the full range of changes that occur in gB during fusion. It is also possible that the altered pre-fusion structure of FFWO gB relative to wild type may facilitate interactions between gB and gD or gH-gL during fusion. These possibilities need to be pursued experimentally.

The pre-fusion form of HSV gB present in three different strains, HSV-1 KOS and ANG path and HSV-2 333 undergoes conformational change in response to low pH [8] and this study). The structure of the HSV-1 gB ectodomain truncated at residue 730 has striking structural homology to the low pH, post-fusion form of vesicular stomatitis virus (VSV) G glycoprotein [20,21]. The available gB structure is the post-fusion form [20,22,23]. Whether this form is crystallized at neutral or acidic pH, the structure is essentially identical [20], suggesting that low pH has a negligible effect on truncated gB that already resembles an activated, post-fusion conformation. The pre-fusion x-ray structure of herpes gB is not currently known, but the pre-fusion structure of G at neutral pH has been determined [24]. We propose that the pH-induced transition from pre- to post-fusion gB during membrane fusion is similar to G, which undergoes significant domain rearrangement. There are unique features of the regulation and execution of herpes fusion due to the multiple cellular triggers and multiple viral proteins, however. For example, low pH induces gB to become a lower-order oligomer [8], but acid causes a tighter, stable association of G subunits [25]. Finally, it remains to be seen whether pH-independent entry via penetration at the plasma membrane [26] is accompanied by similar changes in gB conformation.

## Conclusions

Highly fusogenic gB with FFWO activity and wild type gB undergo pH-triggered changes in antigenic conformation and oligomeric structure. The structure of gB is not globally altered. The mutant, FFWO gB may have a pre-fusion conformation that facilitates membrane fusion, but it may be triggered by low pH in a manner similar to wild type. Entry of a FFWO strain of HSV is inactivated by acid pH. Low pH-triggered changes in gB are independent of the UL45 protein. The available data support a model in which a cellular cue, such as endosomal low pH, triggers structural changes in gB that are critical for fusion and entry.

## Methods

### Cells and viruses

Vero cells (American Type Culture Collection [ATCC], Rockville, MD) were propagated in Dulbecco's modified Eagle's medium (DMEM; Invitrogen, Grand Island, NY) supplemented with 10% fetal bovine serum (FBS; Gemini Bio-Products, West Sacramento, CA). HSV-1 strains ANG path, KOS, and KOS UL45 D [9] were propagated and titered on Vero cells.

### Antibodies

Mouse monoclonal antibodies (MAbs) to gB designated DL16, SS10 and SS106 [17] and gB-specific rabbit polyclonal antibody R69 were kindly provided by Roselyn Eisenberg and Gary Cohen, University of Pennsylvania. The anti-gB MAbs H126 [27] and H1817 were obtained from Virusys. Rabbit polyclonal sera to HSV-1 UL45 protein was obtained from Curtis Brandt [28].

### Dot blot analysis

Cell-free preparations of extracellular HSV-1 ANG path or KOS strains were diluted in serum-free, bicarbonate-free DMEM with 0.2% BSA and 5 mM each of HEPES (Life Technologies), 2-(*N*-morpholino)ethanesulfonic acid (MES; Sigma), and sodium succinate (Sigma) to achieve final pHs ranging from 7.4 to 5.2. Samples were incubated at 37°C for 5 min. Samples were either blotted directly to nitrocellulose with a Mini Fold dot blot system (Whatman) or were first neutralized by addition of pretitrated amounts of 0.05 N NaOH. In each case, equivalent amounts of ANG path and KOS virions (10^6 ^- 10^7 ^PFU) were blotted based on reactivity of the indicated antibody with virions treated with pH 7.4. Membranes were blocked and incubated at neutral pH with anti-gB monoclonal antibody. After incubation with horseradish peroxidase-conjugated goat-anti-mouse antibody, enhanced chemiluminescent substrate (Pierce) was added, and blots were exposed to X-ray film (Kodak). To highlight reduced reactivity or the pH threshold, exposures in which gB reactivity is in the linear range of detection for a given MAb are shown. Thus, the apparent absence of reactivity does not indicate a complete failure of an antibody to bind.

### Assay for sensitivity of oligomeric gB to detergent

The oligomeric conformation of gB from virions exposed to pH < 6.0 is sensitive to 1% SDS as assessed by "native" PAGE [8]. HSV-1 ANG path or KOS virions (~ 10^5 ^PFU) were treated with medium adjusted to pHs ranging from 7.4 to 5.2 as described above for dot blot. Virions were adjusted to 1% SDS and were then added to polyacrylamide gel electrophoresis (PAGE) sample buffer containing 0.2% sodium dodecyl sulfate (SDS) and no reducing agent ("native" conditions). Samples were not heated and were resolved by PAGE. After transfer to nitrocellulose, membranes were blocked and incubated with rabbit polyclonal antibody specific for gB. After incubation with horseradish peroxidase-conjugated goat-anti-rabbit antibody, enhanced chemiluminescent substrate (Pierce) was added and membranes were exposed to X-ray film (Kodak).

### Inactivation of virions by low pH

HSV-1 ANG path or KOS was buffered in serum-free, sodium bicarbonate-free DMEM containing 0.2% BSA with 5 mM each of HEPES, MES and succinate to achieve final pHs ranging from 7.2 to 4.8 and incubated at 37°C for 5 min. Virions were neutralized to pH 7.4 by addition of pretitrated amounts of 0.05 N NaOH. Samples were diluted in sodium bicarbonate-buffered DMEM (pH 7.6) with 10% fetal bovine serum, and added to Vero cell monolayers for 18 hr. Plaque formation was evaluated by immunoperoxidase staining. Infectivity of samples maintained at pH 7.4 was set to 100%.

## Competing interests

The authors declare that they have no competing interests.

## Authors' contributions

All authors have read and approved the final manuscript. CRS, SJD, and AVN carried out experiments and AVN wrote the manuscript.
